# Divergent Selection on Opsins Drives Incipient Speciation in Lake Victoria Cichlids

**DOI:** 10.1371/journal.pbio.0040433

**Published:** 2006-12-05

**Authors:** Yohey Terai, Ole Seehausen, Takeshi Sasaki, Kazuhiko Takahashi, Shinji Mizoiri, Tohru Sugawara, Tetsu Sato, Masakatsu Watanabe, Nellie Konijnendijk, Hillary D. J Mrosso, Hidenori Tachida, Hiroo Imai, Yoshinori Shichida, Norihiro Okada

**Affiliations:** 1 Graduate School of Bioscience and Biotechnology, Tokyo Institute of Technology, Yokohama, Japan; 2 Department of Aquatic Ecology and Evolution, Institute of Zoology, University of Bern, Bern, Switzerland; 3 EAWAG Ecology Center, Swiss Federal Institute for Aquatic Science and Technology, Kastanienbaum, Switzerland; 4 Division of Speciation, National Institute of Basic Biology, Okazaki, Aichi, Japan; 5 Tanzania Fisheries Research Institute, Mwanza Fisheries Research Center, Mwanza, Tanzania; 6 Department of Biology, Faculty of Sciences, Kyushu University, Fukuoka, Japan; 7 Department of Biophysics, Graduate School of Science, Kyoto University, Japan; 8 Core Research for Evolutional Science and Technology, Japan Science and Technology Corporation, Kyoto, Japan; University of Edinburgh, United Kingdom

## Abstract

Divergent natural selection acting on ecological traits, which also affect mate choice, is a key element of ecological speciation theory, but has not previously been demonstrated at the molecular gene level to our knowledge. Here we demonstrate parallel evolution in two cichlid genera under strong divergent selection in a gene that affects both. Strong divergent natural selection fixed opsin proteins with different predicted light absorbance properties at opposite ends of an environmental gradient. By expressing them and measuring absorbance, we show that the reciprocal fixation adapts populations to divergent light environments. The divergent evolution of the visual system coincides with divergence in male breeding coloration, consistent with incipient ecological by-product speciation.

## Abstract 2

The rapid evolution of African cichlid fish driven by strong divergent selection is revealed in a gene that is responsible for ecological adaptation. This gene also affects mate choice and is thus consistent with incipient ecological by-product speciation.

## Introduction

Adaptive radiation, the generation of ecological diversity in a rapidly multiplying lineage, is receiving much attention by evolutionary biologists and ecologists because it is thought to be a force able to quickly generate large numbers of new species and ecological diversity through multiple episodes of ecological speciation [1]. Divergent natural selection (selection on ecologically relevant traits that favors different alleles in different environments) is thought to be its main driver [1,2]. It can potentially cause ecological differentiation and speciation simultaneously when its action on ecological traits in contrasting environments affects mate choice as a by-product [1]. The concept of by-product speciation is theoretically and mathematically well established [1,3], and experimental evidence supports that the mechanism works [2,4,5]. However, because the genes responsible for variation in ecological and mating traits are rarely identified, the hypothesis has remained largely untested at the gene level. In particular, that selection has fixed alleles with opposite effects on an adaptive trait in different closely related populations has rarely been demonstrated [6,7], and to our knowledge, not for traits that affect adaptation and mating preferences simultaneously, even though chromosomal regions with effects on both have been singled out by quantitative trait locus mapping [4,5]. Yet, without such demonstration, the discussion about the role of divergent natural selection in speciation remains somewhat speculative.

African cichlid fish are becoming a model system for the genetics of vertebrate speciation and adaptive radiation [8]. Lake Victoria, the largest of the African great lakes, hosts the youngest of the large cichlid radiations. Geological evidence even suggests that Lake Victoria dried up at the end of the Pleistocene and refilled only 15,000 years ago [9]. Levels of polymorphism in mitochondrial DNA suggest that the neutral genetic diversity contained in the radiation is less than 200,000 y old [10] and may indeed be younger [11]. Surprisingly, despite 10-fold lower mitochondrial DNA diversity, the phenotypic diversity among Lake Victoria cichlids is similar to that in the several-million-y-old-Lake Malawi [12]. This implies rapid selection-driven diversification in Lake Victoria but remained untested at the gene level.

Male nuptial coloration is one of the most amazingly variable phenotypic traits among Lake Victoria cichlid fish. Water is a dense medium that generates highly heterogeneous light environments that fish have to adapt their visual systems to. Visual pigments in the photoreceptor cells of the retina consist of a light-absorbing component—the chromophore—and a protein moiety, the opsin [13]. The light sensitivity of a visual pigment is determined by the chromophore (A1 [11-*cis*-retinal] or A2 [11-*cis*-3, 4-dehydroretinal] pigments) and by its interaction with the amino acid residues coating the retinal binding pocket of the opsin in which the chromophore lies [14].

In haplochromine cichlids, eight different opsins have been found [15–18]. As predicted by adaptive evolution, the most variable between species are those that absorb at the extreme ends of the light spectrum where most environmental variation in light transmission is found: short wavelength–sensitive opsin 1 and long wavelength–sensitive opsin *(LWS)* [17].

Within Lake Victoria, *LWS,* which has a peak value of light absorption (λ_max_) at long (red) wavelengths [16], is by far the most variable opsin [17]. *LWS* is a candidate for a gene under strong divergent selection in Lake Victoria, because it entails five times more variation in the cichlids of Lake Victoria than in the at least 10-fold older Lake Malawi cichlid radiation [19,20]. The photic environment in Lake Victoria is characterized by much steeper gradients in water clarity, light intensity, and spectral composition [21]. Given the importance of vision in food acquisition, predator avoidance, territorial defence, and mate choice of cichlid fish, these gradients are likely exerting strong selection on opsin genes and on associated mating traits. In several pairs of closely related species of Lake Victoria cichlids, the species with the larger LWS λ_max_ has red male breeding colors and lower detection thresholds for red light, whereas the species with smaller LWS λ_max_ has blue breeding colors and is less sensitive to red light and more sensitive to blue light, consistent with sensory drive (divergence in male coloration evolving as a consequence of divergent visual sensitivities) [20,22,23]. Hence, the *LWS* gene may be involved in ecological adaptation and mate choice simultaneously. Here we demonstrate evolution in *LWS* under strong divergent ecological selection along an environmental gradient, and we show correlated divergence in male nuptial coloration, implicating population divergence through sensory drive.

## Results/Discussion

### Population Divergence at the *LWS* Gene

**Figure 1:**
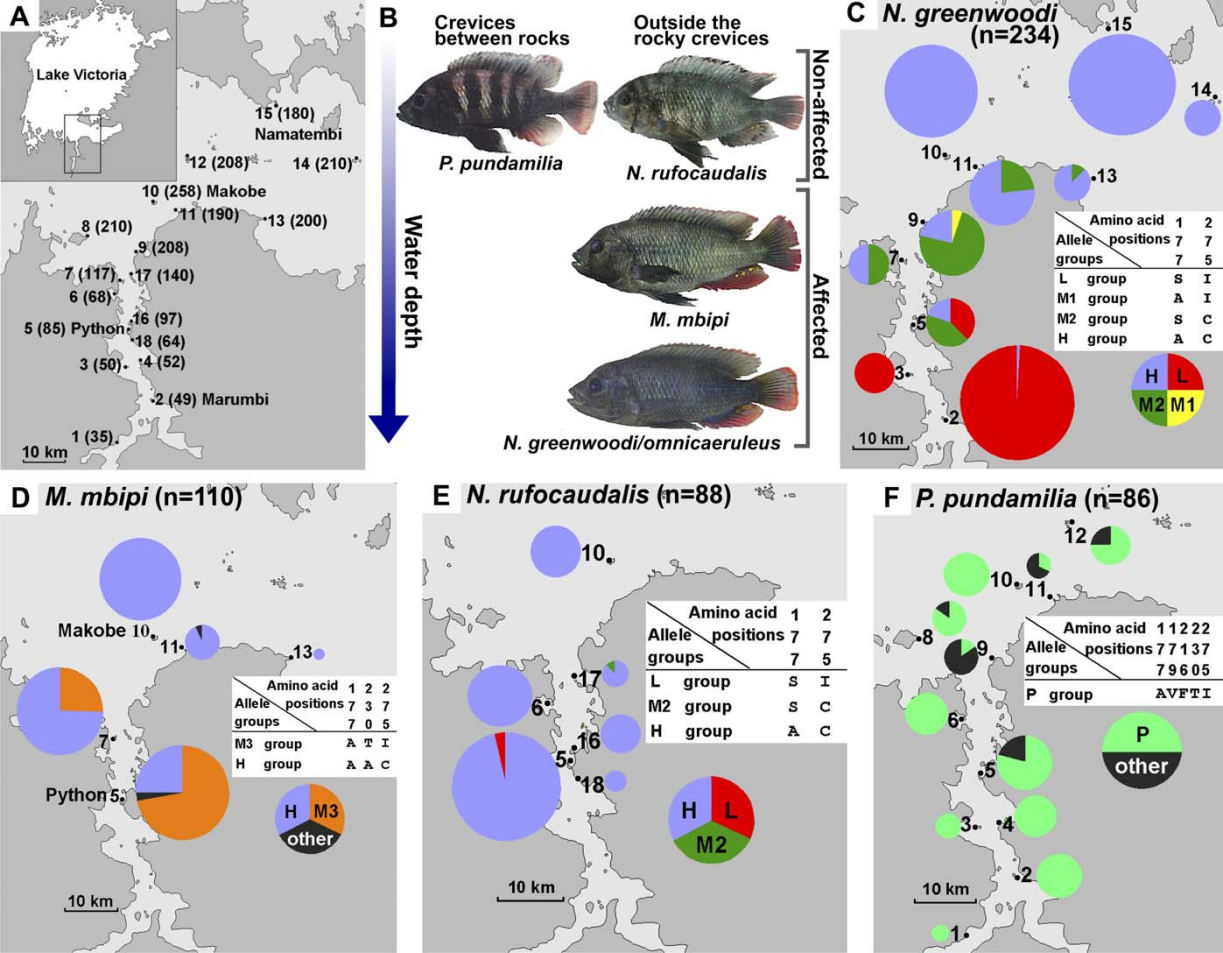
Maps and Frequencies of *LWS* Alleles (A) The study area in southern Lake Victoria. Arabic numerals indicate stations at which cichlids were collected. The Secchi disk water transparency (cm) at each station is shown in parentheses. The stations are: 1, Buyago Rocks; 2, Marumbi Island; 3, Matumbi Island; 4, Luanso Island; 5, Python (Nyamatala) Islands; 6, Kissenda Island; 7, Hippo Island; 8, Juma Island; 9, Bwiru Point; 10, Makobe Island; 11, Igombe Island; 12, Ruti Island; 13, Senga Point; 14, Sozihe Islands; 15, Namatembi Island; 16, Nyamatala (Nyameruguyu) Island; 17, Gabalema Islands; 18, north end of Luanso Bay. Where the local name differs from that used in previous publications, the local name is indicated in parenthesis. (B) A representation of the microhabitat distribution of the studied species by water depth and between, as opposed to outside, the rocky boulders. Photos show males of the blue morph in nuptial coloration. “Affected” and “Non-affected” indicate the species with the *LWS* allele frequencies that are strongly affected and not strongly affected by variation in water transparency, respectively. (C) *LWS* allele group frequencies in the populations of *N. greenwoodi/omnicaeruleus*. (D) *LWS* allele group frequencies in the populations of M. mbipi. (E) *LWS* allele group frequencies in the populations of N. rufocaudalis. (F) *LWS* allele group frequencies in the populations of P. pundamilia. In (B–E), Arabic numerals correspond to those in (A). The size of a pie indicates the number of haplotypes sequenced: N. greenwoodi: *n* = 58 at station 2; *n* = 10 at 3; *n* = 14 at 5; *n* = 10 at 7; *n* = 14 at 9; *n* = 22 at 11; *n* = 8 at 13; *n* = 8 at 14; *n* = 50 at 15, and *N. omnicaeruleus, n* = 40 at 10. M. mbipi: *n* = 36 at station 5; *n* = 32 at 7; *n* = 30 at 10; *n* = 10 at 11; *n* = 2 at 13. N. rufocaudalis: *n* = 42 at station 5; *n* = 16 at 6; *n* = 10 at 10; *n* = 10 at 16; *n* = 6 at 17; *n* = 4 at 18. P. pundamilia: *n* = 2 at station 1; *n* = 10 at 2; *n* = 4 at 3; *n* = 8 at 4; *n* = 18 at 5; *n* = 8 at 6; *n* = 6 at 8; *n* = 6 at 9; *n* = 10 at 10; *n* = 6 at 11; *n* = 8 at 12. The color of the sections of the pie indicates the frequency of allele groups L (red), M1 (yellow), M2 (green), H (blue), M3 (orange), P (blue-green), and other alleles (black). The amino acid differences among allele groups are shown for every species in the corresponding white panels.

We studied multiple populations of each of four different Lake Victoria cichlid species along a 100-km-long cline in water clarity (Figure 1A). All four have lake-wide but patchy distributions, being restricted to rocky islands and headlands: Neochromis greenwoodi (including its offshore incipient species N. omnicaeruleus [24]), *Neochromis rufocaudalis, Mbipia mbipi,* and Pundamilia pundamilia [24]. We determined the sequences of exons 2–5 of *LWS* (872 base pairs [bp]) that code the trans-membrane region from 117 individuals (234 alleles) of N. greenwoodi/*omnicaeruleus* (10 populations), 44 individuals (88 alleles) of N. rufocaudalis (6 populations), 55 individuals (110 alleles) of Mbipia mbipi (5 populations), and 43 individuals (86 alleles) of Pundamilia pundamilia (11 populations). For this analysis, we considered it appropriate to treat *N. greenwoodi/omnicaeruleus* as one taxon (i.e., superspecies), because N. omnicaeruleus replaces N. greenwoodi at the clear water islands in the Speke Gulf and differs only in male coloration (light blue) from neighbouring N. greenwoodi populations along the mainland (blue-black) [24]. The four taxa occupy different water depths and microhabitats, living either mostly outside or inside the rocky interstices (Figures 1B and S1). N. rufocaudalis and P. pundamilia are everywhere restricted to very shallow waters. N. rufocaudalis grazes on algae on the outside of rocks, whereas P. pundamilia lives in crevices between rocks, feeding on insect larvae. M. mbipi has a deeper modal depth and lives predominantly outside the rocky crevices. *N. greenwoodi/omnicaeruleus* has the widest depth range, a modal depth slightly deeper than *M. mbipi*, and also lives mostly outside the rocky crevices [25] (Figures 1B and S1). Both are omnivorous feeders. We predicted that *N. greenwoodi/omnicaeruleus* and M. mbipi should experience strong divergent selection on the visual system between populations, exerted by differences in light transmission between islands with different water clarity, whereas species that live exclusively in very shallow water (and in rocky interstices) would be less affected.

We report the results on *N. greenwoodi/omnicaeruleus* first and then compare results on the three other species against them. We observed ten polymorphic sites (one synonymous, nine nonsynonymous) among the *LWS* sequences of *N. greenwoodi/omnicaeruleus* (Figure S2). From the bovine rhodopsin crystal structure [26], we inferred that two of the variable amino acid positions, 177 and 275, are located in the retinal binding pocket, and the others are distant from retinal. We focused on positions 177 (nucleotide site 529) and 275 (sites 823 and 824) and divided all observed alleles into four groups based on these two amino acid positions: The L group includes all alleles with 177S (529T) and 275I (823A and 824T). The H group includes all alleles with 177A (529G) and 275C (823T and 824G). The M1 group includes all alleles with 177A (529G) but 275I (823A and 824T). The M2 group includes all alleles with 177S (529T) but 275C (823T and 824G). M1 and M2 alleles can be considered recombinants of L and H alleles or intermediate alleles. The frequencies of allele groups in the ten populations are shown in Figure 1C.

In populations that live in very turbid water (<80 cm Secchi disk transparency), the L group alleles appeared to be fixed or almost fixed (station 2: number of L alleles [L] = 57, 98.3% and station 3: L = 10, 100%) (Figures 1C and S2). On the other hand, the H group alleles (H) appeared to be fixed in all the offshore island populations with high water transparency (≥180 cm; station 14: H = 8, 100%, station 15: H = 50, 100%, station 10: H = 40, 100%) (Figures 1C and S2). With one exception (one H group allele at station 2), the L and H allele groups were observed only in populations that live in waters of equal to or less than, and equal to or more than 85 cm Secchi transparency, respectively (Figure 1C). Mantel tests [27] of the significance of correlations between matrices of (i) pairwise population differentiation at the *LWS* locus, (ii) pairwise difference in water transparency, and (iii) pairwise geographical distance, revealed a highly significant positive correlation between *LWS* divergence and transparency (cross matrix correlation 0.80, *p* = 0.0008), but only a weak correlation between *LWS* divergence and geographical distance (0.55, *p* = 0.04), and an even weaker correlation between transparency and geographical distance (0.42, *p* = 0.08). The clinal change from L-dominated to H-dominated populations through populations dominated by the recombinant/intermediate allele groups M1 and M2 is consistent with gene flow between populations in a stepping stone fashion. We estimated the number of migrants per generation (M) from neutral marker F_ST_ (Table S1) [M = (1/ F_ST −_ −1)/4] as M = 5.6 between the most turbid inshore station 2 and the most clear offshore station 10, M = 7.6 between station 2 and the offshore Namatembi Island (station 15), and M = 14.5 between the two clear offshore stations 10 and 15. However, F_ST_-derived estimates of M should be taken with great caution because they rely on the assumptions of Wright's island model [28] (all populations have the same population size and migration rate, are in migration/drift equilibrium, equally likely to give and receive migrants from all other populations, and the number of alleles is infinite) [29]. Our estimates of gene flow between the most distant and the most environmentally distinct islands may be taken as a clear indication that significant differentiation at the *LWS* locus between any two geographically closer populations on our transect requires divergent selection on *LWS* to overcome gene flow.

### Divergent Selection Acting on *LWS* Gene

**Figure 2:**
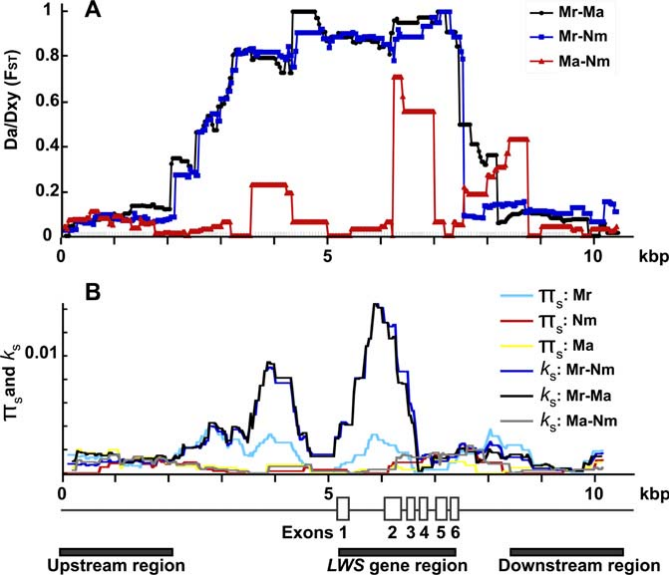
Detection of Selection Pressure on the *LWS* Gene The genome structure of the *LWS* gene and its flanking region and (A) sliding-window analysis of F_ST_ between Marumbi (Mr., station 2: N. greenwoodi), Namatembi (Nm., station 15: N. greenwoodi), and Makobe (Ma., station 10: N. omnicaeruleus) populations (Marumbi versus Makobe, Marumbi versus Namatembi, and Makobe versus Namatembi indicated by black, blue, and red lines, respectively). (B) Sliding-window analysis of silent polymorphism (π_s_) in Marumbi (Mr: light blue), Namatembi (Nm: red), and Makobe (Ma: yellow), and silent divergence *(k_s_)* between Marumbi, Namatembi, and Makobe populations (Marumbi versus Namatembi, Marumbi versus Makobe, and Makobe versus Namatembi indicated by blue, black, and gray lines, respectively). π _s_ and *k*_s_ were calculated for segments of 700 bp in 25-bp intervals. The solid lines under the genome structure of *LWS* indicate the three regions used in HKA tests (Table S2). The *LWS* gene region is defined as the sequence between initiation codon and stop codon (2205 bp) of *LWS*. The up- and downstream regions are defined as the 5′ and 3′ ends of sequences of the same length as the *LWS* gene region.

To search for the molecular signature of divergent natural selection between populations, we analyzed sequences up- and downstream of *LWS*. The DNA fragments including the *LWS* gene and its 5-kilobase (kb) upstream and 3.5-kb downstream flanking sequences (total 11 kb) were amplified by long PCR, and 10.5 kb were determined. We sequenced 18 alleles (from nine individuals of *N. greenwoodi/omnicaeruleus*) from each of the Marumbi (station 2), Makobe (station 10), and Namatembi (station 15) populations. Sliding window analysis revealed that population differentiation in the *LWS* gene region between populations from clear and turbid waters (F_ST_ > 0.8) (Figure 2A, Makobe-Marumbi, black line; Namatembi-Marumbi, blue line) was at least four times larger than in the regions of up- and downstream sequences (F_ST_ < 0.2) and 17 times larger than in unlinked neutral polymorphic loci (F_ST_ < 0.046: Table S1). Populations in clear and turbid waters are strongly differentiated from one another only in the sequences of the *LWS* gene region. The contrast between differentiation in *LWS* gene region versus flanking sequences was shown to be statistically significant by applying the McDonald test [30] (Makobe: *p* < 0.0001, Namatembi: *p* < 0.0001, Marumbi: *p* < 0.024, with the recombination parameter set to 2, 4, 10, 32, and 1000 replicates) and was further supported by HKA tests [31] revealing a significantly smaller ratio of within-population polymorphism to between-population divergence in the *LWS* gene region than in up- and downstream flanking regions (Table S2). The larger F_ST_ value can be caused either by stronger differentiation between populations or by reduced variation within populations [32]. In the present case, the differentiation is caused by larger divergence of the region between exon 1 and exon 3 compared to those in other regions (Figure 2B, Makobe-Marumbi, black line; Namatembi-Marumbi, blue line). This result strongly suggests action of divergent selection on the *LWS* gene, possibly caused by the difference between clear and turbid waters.

In contrast, only weak population differentiation was observed throughout the sequences when we compared populations from similar water transparencies (Figure 2A, red line, Figure 2B, gray line: Makobe-Namatembi), and no evidence for divergent selection (HKA tests, Table S2). Hence, divergent selection has acted on the *LWS* alleles only between populations from different water transparencies, strongly implicating divergent adaptation to the different photic environments. The F_ST_ values in the *LWS* gene region imply fewer than one migrant every ten generations (<0.06 per generation), compared to about one migrant per generation in the up- and downstream regions, and six to eight at unlinked neutral loci. Hence, strong divergent selection effectively reduces migration in the *LWS* region compared to the rest of the genome by more than an order of magnitude. The F_ST_ values in 2–3 kb of sequences upstream of *LWS* were also higher than further up- and downstream, suggesting the possibility of genetic hitchhiking caused by the divergent selection on the downstream *LWS* gene, or further divergent selection on this region itself (Figure 2A, black and blue lines).

### Parallel Divergent Adaptation of the *LWS* Gene

**Figure 3:**
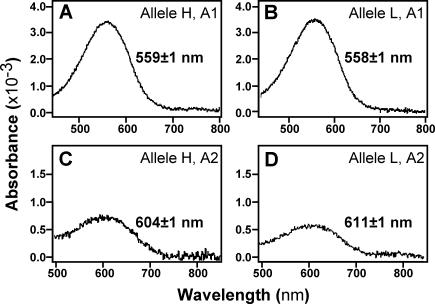
Absorption Spectra of the LWS Pigments Evaluated by the Dark–Light Difference Spectra The LWS pigments were reconstituted from (A) H allele with A1 retinal, (B) L allele with A1 retinal, (C) H allele with A2 retinal, and (D) L allele with A2 retinal. The λ_max_ values are indicated with their standard errors.

To determine the adaptive significance of the *LWS* divergence, we reconstituted the LWS pigments from both L and H alleles in vitro with both A1- and A2-derived retinal and measured their absorption spectra. The ratio of A1 to A2 chromophores in haplochromine cichlids was reported to be about ten to one [16]. We confirmed this ratio from laboratory-bred Lake Victoria cichlids and detected both chromophores in N. greenwoodi from the Mwanza Gulf by the method described previously [16]. Replacing an A1- by an A2-derived retinal shifts the λ_max_ value to a longer wavelength [33]. Even though in other vertebrates a 7-nm shift was reported even with A1 pigments for the amino acid replacement at the position corresponding to cichlid LWS position 177 (position 180 in human red and green pigments [14]), we did not observe any difference between A1 pigments of H and L alleles (Figure 3A and 3B). However, we found that the peak spectral sensitivity (λ_max_) of the A2 pigment of the L allele was red shifted by 7 nm compared to that of the H allele (Figure 3C and 3D).

The transmission light spectra at different water transparencies in Lake Victoria were measured previously. Highly transparent waters transmit broad spectra, whereas the dissolved and dispersed organic matter in turbid waters selectively scatters and absorbs light of short wavelengths, leading to a shift in spectral composition towards longer wavelengths [21]. Although we do not rule out other functional differences between H and L alleles [13], the fixation in red-shifted turbid waters of the *LWS* alleles of the L group, from which we reconstituted a red shifted A2 pigment, is likely an adaptation to a photic environment enriched in longer wavelengths.

Our results on the three other species corroborate this conclusion. In M. mbipi, the species that is ecologically most similar to N. greenwoodi (Figures 1B and S1), populations from high (258 cm Secchi, station 10, *n* = 30 alleles) and low (85 cm Secchi, station 5, *n* = 36 alleles) transparency were strongly differentiated in their *LWS* sequences too (Figure 1D, F_ST_ = 0.54, *p* < 0.001). Just like in N. greenwoodi, the clear water population of M. mbipi was fixed for H group alleles. Going into more turbid waters, the frequencies of these alleles stayed somewhat higher than in the somewhat deeper living sympatric N. greenwoodi. In contrast with N. greenwoodi, the M. mbipi population from the most turbid water in which this species was found (station 5) was dominated by a group of M3 alleles including all alleles with amino acid positions 177A (529G), 230T (688A), and 275I (823A and 824T) (Figures 1D and S3).

In N. rufocaudalis, a species that lives exclusively in very shallow waters (Figures 1B and S1), the allele of the H group that in the deeper living N. greenwoodi and M. mbipi dominated only in clear water populations dominated in all populations. One allele of the M2 group was found at a place of intermediate transparency, and the only two alleles of the L group came from the turbid end of this species' distribution range (Figures 1E and S4). In the cave-dwelling *P. pundamilia,* populations from high and low water transparency were identical and a different allele group P (including all alleles with 177A, 179V, 216F, 230T, and 275I) dominated (Figures 1F and S5). Hence, (i) strong divergent evolution at the *LWS* locus between populations occurred in two species from photic habitats that are strongly affected by variation in water transparency, but not in two species whose habitats are not strongly affected; (ii) the transparency-associated geographical cline in allele frequencies observed within two species was mirrored by a depth-associated cline in allele frequencies between the three species—*N. rufocaudalis, M. mbipi,* and N. greenwoodi—that live outside the rocky crevices (Figure 1B). The parallel divergent adaptation in N. greenwoodi and M. mbipi is likely to include some true parallel evolution at the gene level, because visual adaptation to turbid waters is achieved by substituting the clear water *LWS* allele H with different alleles in the two species (L and M3, Figure S6), which cannot be explained by very recent introgressive hybridization.

### Correlation between Frequencies of *LWS* Allele and Male Nuptial Coloration

Nuptial color display is important in mate choice of Lake Victoria cichlids [21,34,35]. Female mating preferences may be affected by color vision [22,36], and closely related species with red versus blue male nuptial coloration possess *LWS* alleles with larger and smaller λ_max_ respectively, where the difference in λ_max_ is similar to the one observed here between L and H alleles [20]. It is therefore conceivable that the reciprocal fixation of *LWS* alleles with small and large λ_max_ would cause populations at the ends of the water transparency cline to diverge in nuptial coloration, a first step towards parapatric ecological by-product speciation [37]. We collected and photographed a large number of males in breeding dress from all populations of *N. greenwoodi/omnicaeruleus* and M. mbipi and determined the frequencies of male nuptial color morphs in each population. Globally, most males of these species are blue or blue-black, but yellow-red *(N. greenwoodi/omnicaeruleus)* or yellow *(M. mbipi)* males occur at low, but variable frequencies in much of their ranges. In the relatively turbid waters of Lake Victoria, yellow and red light travel further than blue light, and yellow and red colors may hence be perceived as brighter than blue at long path length (deeper water) [22]. This effect is stronger the more turbid the water [21].

**Figure 4:**
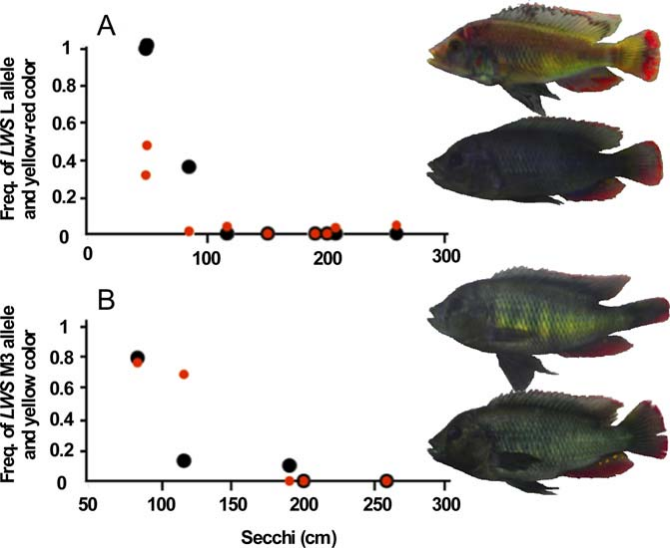
The Relationship between the Frequency of *LWS* Alleles, Male Nuptial Color Morphs, and Water Transparency (A) The relationship between the frequency of long wavelength–sensitive allele group L at the *LWS* locus (black), yellow-red male nuptial color (red), and water transparency (Secchi disk [cm]) in N. greenwoodi. (B) The relationship between the frequency of allele group M3 at the *LWS* locus (black), yellow male nuptial color (red), and water transparency in M. mbipi.

In populations of *N. greenwoodi/omnicaeruleus,* the frequency of yellow-red males ranged from 0%–47% (Figure 4A) and was strongly positively correlated with the frequency of relatively longer wavelength–shifted *LWS* alleles (cross matrix correlation between population differentiation at the *LWS* locus and in color morph frequency 0.87, Mantel test *p* = 0.019), and less strongly with water transparency (cross matrix correlation 0.58, *p* = 0.023). Both relationships were also strong in M. mbipi (Figure 4B, cross matrix correlation between difference in the frequency of yellow males and differentiation at the *LWS* locus 0.83, *p <* 0.001, with water transparency 0.93, *p <* 0.001), and populations at the extremes of the cline were almost fixed for the different colors. Hence we have evidence for parallel correlated divergent evolution in a visual gene and in male nuptial coloration in two species along the same environmental cline. Divergence in male nuptial coloration may be a consequence of divergent adaptation in the *LWS* gene. Although long wave–shifted male nuptial coloration became common, it has not become fixed in populations in which long wave–shifted *LWS* alleles got fixed. This may suggest that population divergence at the *LWS* locus alone is not necessarily sufficient for complete population divergence in male nuptial coloration. This may not be surprising, because several selection forces are known to operate on male nuptial coloration in haplochromine cichlids [21,34,35,38]. Our data suggest that evolution at opsin genes may influence the balance between these. It is possible that complete fixation of alternative nuptial colors requires reinforcement of mating preferences in sympatry, something we are currently testing with another data set.

The parallel divergence in *LWS* along the same environmental cline in two unrelated cichlid species suggests rapid divergent evolution under natural and sexual selection, may not be uncommon along the strong gradients in light environment that characterize Lake Victoria. The nucleotides in L, H, and M3 alleles at 529, 688, 823, and 824 also occur in other cichlid species in Lake Victoria [19], and the different alleles are not monophyletic within the species that we studied here (Figure S6). This suggests that the alleles originated prior to establishment of the species, and certainly prior to establishment of the intraspecific population structure that we investigated here, consistent with the fact that the island populations that we sampled cannot be older than 15,000 y independent of whether the lake was entirely dry during its last major low stand [9]. Hence, the intraspecific allelic diversity must have been acquired either from a single polymorphic ancestral population or through hybridization between species. Persistence of L/H allele polymorphisms seems rare in contemporaneous populations, and we found no evidence for L/M3 polymorphisms at all (Figures 1 and S6). It seems therefore more likely that locally favored *LWS* alleles are acquired by occasional hybridization in overlap zones between species adapted to different light environments (i.e., different water depths), a hypothesis that is consistent with our finding that the most common H group alleles in clear water populations of the unrelated species *M. mbipi, N. greenwoodi,* and N. rufocaudalis are sequence-identical (Figure S6). It is also consistent with the observation that *P. pundamilia,* the species with quite different habitat use, shares none of the *LWS* alleles with the other species.

Here, we have for the first time, to our knowledge, shown rapid evolution in African cichlid fish driven by strong divergent selection in a gene responsible for ecological adaptation, which also affects mate choice, consistent with incipient ecological by-product speciation that occurs in parallel in several unrelated species. The evolutionary history of the mutations involved, and the relevance of reticulate evolution in swapping locally advantageous mutations between species during the rapid adaptive radiation of Lake Victoria cichlids deserve the attention of evolutionary geneticists in the future.

## Materials and Methods

### Samples.

Specimens of *N. greenwoodi, N. omnicaeruleus, M. mbipi,* and P. pundamilia were collected by OS in 1995 and 1996, except for 17 of the 25 individuals from Namatembi, which were collected in 2005 by TS, HM, and SM, 11 of 18 of M. mbipi from Python, and 12 of 16 from Hippo Island, which were collected in 2005 by NK and HM, and eight of 15 M. mbipi from Makobe, which were collected in 2004 by HM, SM, and S Mzighani. Specimens of N. rufocaudalis were collected in 2004 and 2005 by SM, KT, and S. Mzighani. Identification of all specimens was verified by OS.

### DNA sequencing.

Determination of the cichlid *LWS* gene was as described [19]. To determine the *LWS* flanking sequences, the BAC clone including the *LWS* gene was screened and isolated from a Lake Victoria cichlid BAC library [39]. The DNA of BAC clone was digested with *Hin*dIII and subsequently subcloned into pUC 19 plasmid vector. The *Hin*dIII fragment including the *LWS* gene was screened, and the sequence determined by the shot-gun sequencing method described elsewhere [40]. Based on the sequence of the *Hin*dIII fragment, we designed primers for PCR amplification and performed direct sequencing. The positions of primers are described above and below the schematic *Hin*dIII fragment (Figure S7). The DNA fragment including *LWS* gene and both flanking regions was amplified by LA (long and accurate) PCR (TaKaRa, Shiga, Japan) using primers LWSB_LF and LWSB_LR with genomic DNA (∼50 ng) as templates. Amplifications were carried out in the PTC-100 Programmable Thermal Controller (MJ Research, Massachusetts, United States). The PCR program consisted of a denaturation step for 3 min at 94 °C, followed by 30 cycles, each cycle consisting of 20 s denaturation at 98 °C, 15 min annealing and extension at 68 °C. The amplification product was then used as a template to amplify and sequence four overlapping fragments using the primers for upstream (LWSB_LF and LWSB_R3), (LWSB_F3 and LWSB_R5), *LWS* gene (LWSB_F5 and LWSB_R8), and downstream (LWSB_F8 and LWSB_LR) regions. These PCR products were purified and determined by direct sequencing with all primers described above and below the schematic *Hin*dIII fragment (Figure S7). Once determined, the sequences were connected by using the GENETYX-MAC Version 10.1 program. The sequences of primers are described in Figure S8.

### Measurement of absorption spectra of cichlid *LWS* pigments.

The H allele sequence was amplified via RT-PCR using total RNA extracted from eyes of Lake Victoria cichlid as template with a pair of PCR primers (Eco6*LWS* and *LWS*_Flag_stop_NotI, Figure S8) designed to produce fusion protein with FLAG-tag (Sigma-Aldrich, St. Louis, Missouri, United States) in its C terminus. The amplified DNA fragments were digested with *Eco*RI and *Not*I and cloned into the *Eco*RI/*Not*I-digested (removing ID4) pMT5 expression vector [41]. In vitro mutagenesis of the *LWS* for construction of other sequences of alleles, expression, reconstitution, purification, and measurement were performed as described previously [16] with minor modifications. After pigment reconstitution, these experiments were performed under infrared light (>900 nm) with the vision of a digital video-camera in “night shot” mode (SONY, Tokyo, Japan) or complete darkness.

### Matrix correlations.

We calculated the mean pairwise *LWS* sequence distance between populations as *D_xy_* values (the average number of nucleotide substitutions per site between populations) using only the sites that were used for the identification of allele groups. We measured geographical distance as the shortest waterway distance between sites. We calculated the pairwise difference in water transparency and in the frequency of yellow-red or yellow males between sites. To evaluate the significance of correlations between these variables we used Mantel tests [27].

### Population genetic analysis.

The DNA fragment including the *LWS* gene and its flanking sequence (10560 bp) was split into two putative alleles for the analysis by the program DnaSP 4.0 [42]. We performed sliding-window analysis to calculate approximate values for F_ST_ (*D*_a_/*D*_xy_ [43]), π_s_ , and *k*_s_ in 700 bp windows, sliding the window in steps of 25 bp throughout the total 10.5-kb sequences. We performed the McDonald test for heterogeneity across a region of a DNA sequence in the ratio of polymorphism to divergence [30].

## Supporting Information

Figure S1 Habitat Distribution of the Four Species The distribution of (A) *P. pundamilia,* (B) *N. rufocaudalis,* (C) *M. mbipi,* and (D) N. greenwoodi studied along a cline of water transparency (*x*-axis) and water depth (*y*-axis). Arrowheads indicate transparencies at which we sampled. Ranges of occurrence are hatched.
(231 KB PDF)

Figure S2 An Alignment of All Polymorphic Sites in the *LWS* Sequences of N. greenwoodi and N. omnicaeruleus
The nucleotide sites and amino acid positions are shown on top and below the alignment, respectively. Dots indicate where nucleotides are identical with those in the top line. The nucleotide sites 529, 823, and 824 are highlighted. Amino acids in the row directly under the amino acid positions indicate the amino acids translated from the sequence in the top line. Amino acids in the bottom line indicate amino acids substituted from the top line. The sampling station numbers are described in Figure 1A.
(294 KB PDF)

Figure S3 An Alignment of All Polymorphic Sites in 55 *LWS* Sequences from M. mbipi
The nucleotide sites are shown on top of the alignment. n and s indicate nonsynonymous and synonymous sites, respectively. Dots indicate where nucleotides are identical with those in the top line. The sequences of N. omnicaeruleus from Makobe (N. omnicaeruleus Ma) and N. greenwoodi from Marumbi (N. greenwoodi Mr) are aligned at the bottom. The sampling station numbers are described in Figure 1A.
(52 KB PDF)

Figure S4 An Alignment of All Polymorphic Sites in 44 *LWS* Sequences from N. rufocaudalis
The nucleotide sites are shown on top of the alignment. n and s indicate nonsynonymous and synonymous sites, respectively. Dots indicate where nucleotides are identical with those in the top line. The sequences of N. omnicaeruleus from Makobe (N. omnicaeruleus Ma) and N. greenwoodi from Marumbi (N. greenwoodi Mr) are aligned at the bottom. The sampling station numbers are described in Figure 1A.
(50 KB PDF)

Figure S5 An Alignment of All Polymorphic Sites in 43 *LWS* Sequences from P. pundamilia
The nucleotide sites are shown on top of the alignment. n and s indicate nonsynonymous and synonymous sites, respectively. Dots indicate where nucleotides are identical with those in the top line. The sequences of N. omnicaeruleus from Makobe (N. omnicaeruleus Ma) and N. greenwoodi from Marumbi (N. greenwoodi Mr) are aligned at the bottom. The sampling station numbers are described in Figure 1A.
(64 KB PDF)

Figure S6 Maximum-Likelihood Tree for Alleles Studied Here, and Alleles 1–14 [19]. Maximum-likelihood analysis was performed with MOLPHY version 2.3 [44]. An NJ tree was used as the starting tree for a local rearrangement search for the ML tree with the HKY85 model [45]. The scale bar indicates the number of substitutions per site. Bootstrap values are shown at the branches. An alignment of all informative sites of alleles and the frequencies (%) of each allele are shown in right panel. Dots indicate where nucleotides are identical with those in the top line. The frequency of “other” alleles is not shown.
(41 KB PDF)

Figure S7 Schematic Demonstration of the Position of Each Primer on the *LWS* Gene and Its Flanking Region Arrows indicate the primers. The flanking sequences of the *LWS* gene were determined from a BAC clone.
(33 KB PDF)

Figure S8 Sequences of Primers (15 KB PDF)

Table S1 F_ST_ Values for Polymorphic Neutral Markers (81 KB PDF)

Table S2 HKA Test for the Statistical Significance *(p)* of Heterogeneity between Regions (34 KB PDF)

### Accession Numbers

The GenBank http://www.ncbi.nlm.nih.gov/Genbank) accession numbers for DNA sequences discussed in this paper are: AB221133–AB221343 and AB240064–AB240138.

## Abbreviations

A1 chromophore: 11-*cis*-retinal
A2 chromophore: 11-*cis*-3, 4-dehydroretinal
bp: base pair
kb: kilobase
