# Supplementary figures and images for: Divergent Selection on Opsins Drives Incipient Speciation in Lake Victoria Cichlids
Source: PLoS Biol. 2006 Dec 5;4(12):e433. doi: 10.1371/journal.pbio.0040433 (PMC1750929; doi:10.1371/journal.pbio.0040433)

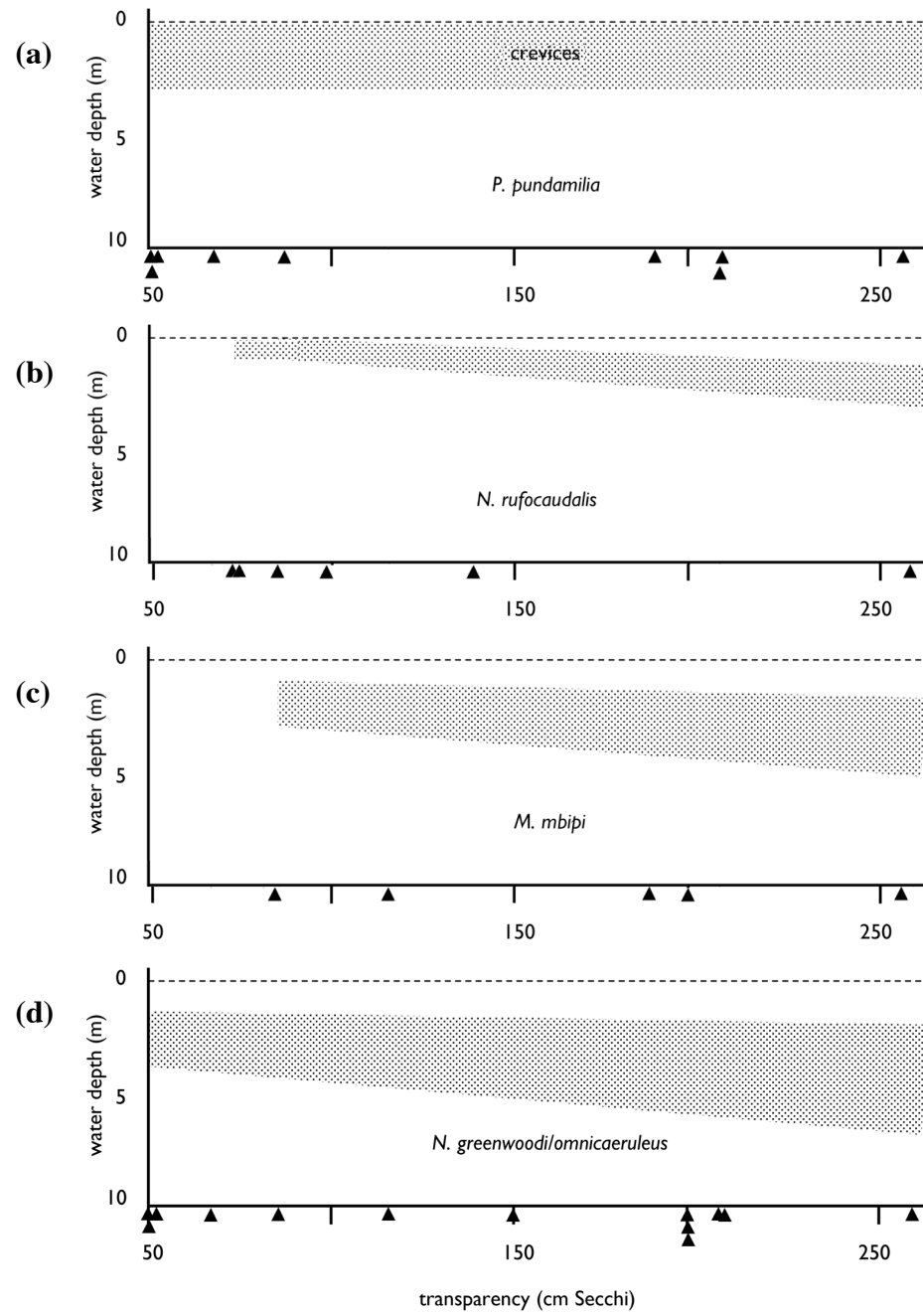

Supplement: Figure S1 — The distribution of (A) P. pundamilia, (B) N. rufocaudalis, (C) M. mbipi, and (D) N. greenwoodi studied along a cline of water transparency (x-axis) and water depth (y-axis). Arrowheads indicate transparencies at which we sampled. Ranges of occurrence are hatched. (231 KB PDF) [file pbio.0040433.sg001.pdf]

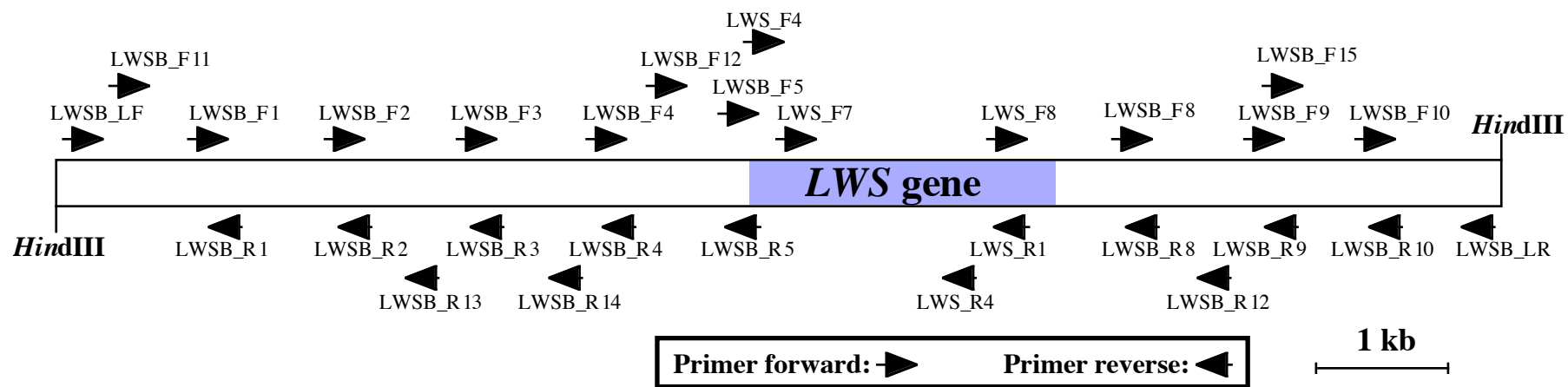

Supplement: Figure S7 — Arrows indicate the primers. The flanking sequences of the LWS gene were determined from a BAC clone. (33 KB PDF) [file pbio.0040433.sg007.pdf]
