# Supplementary material for: Divergent Selection on Opsins Drives Incipient Speciation in Lake Victoria Cichlids
Source: PLoS Biol. 2006 Dec 5;4(12):e433. doi: 10.1371/journal.pbio.0040433 (PMC1750929; doi:10.1371/journal.pbio.0040433)
Supplement: Figure S2 — The nucleotide sites and amino acid positions are shown on top and below the alignment, respectively. Dots indicate where nucleotides are identical with those in the top line. The nucleotide sites 529, 823, and 824 are highlighted. Amino acids in the row directly under the amino acid positions indicate the amino acids translated from the sequence in the top line. Amino acids in the bottom line indicate amino acids substituted from the top line. The sampling station numbers are described in Figure 1A. (294 KB PDF) [file pbio.0040433.sg002.pdf]

nucleotide sites	132	449	465	503	529	535	688	823	824	844	Station	Species	Number of sequences per allele group
syn/non-syn	n	n	s	n	n	n	n	n	n	n			
Mr1	C	T	T	C	T	T	G	A	T	A	(2)	(*N. greenwoodi*)	
Mr16	.	.	.	.	.	.	.	.	.	.	(2)	(*N. greenwoodi*)	
Mr19	.	.	.	.	.	.	.	.	.	.	(2)	(*N. greenwoodi*)	
Mr20	.	.	.	.	.	.	.	.	.	.	(2)	(*N. greenwoodi*)	
Mr21	.	.	.	.	.	.	.	.	.	.	(2)	(*N. greenwoodi*)	
Mr22	.	.	.	.	.	.	.	.	.	.	(2)	(*N. greenwoodi*)	
Mr23	.	.	.	.	.	.	.	.	.	.	(2)	(*N. greenwoodi*)	
Mr24	.	.	.	.	.	.	.	.	.	.	(2)	(*N. greenwoodi*)	
Mr25	.	.	.	.	.	.	.	.	.	.	(2)	(*N. greenwoodi*)	
Mr26	.	.	.	.	.	.	.	.	.	.	(2)	(*N. greenwoodi*)	
Mr27	.	.	.	.	.	.	.	.	.	.	(2)	(*N. greenwoodi*)	
Mr28	.	.	.	.	.	.	.	.	.	.	(2)	(*N. greenwoodi*)	
Mr29	.	.	.	.	.	.	.	.	.	.	(2)	(*N. greenwoodi*)	
Mr3	.	.	.	.	.	.	.	.	.	.	(2)	(*N. greenwoodi*)	L=57
Mr30	.	.	.	.	.	.	.	.	.	.	(2)	(*N. greenwoodi*)	
Mr31	.	.	.	.	.	.	.	.	.	.	(2)	(*N. greenwoodi*)	H=1
Mr4	.	.	.	.	.	.	.	.	.	.	(2)	(*N. greenwoodi*)	
Mr5	.	.	.	.	.	.	.	.	.	.	(2)	(*N. greenwoodi*)	
Mr6	.	.	.	.	K	K	.	W	K	.	(2)	(*N. greenwoodi*)	
Mr7	.	.	.	.	.	.	.	.	.	.	(2)	(*N. greenwoodi*)	
MrVBL1	.	.	.	.	.	.	.	.	.	.	(2)	(*N. greenwoodi*)	
MrVBL2	.	.	.	.	.	.	.	.	.	.	(2)	(*N. greenwoodi*)	
MrVBL3	.	.	.	.	.	.	.	.	.	.	(2)	(*N. greenwoodi*)	
MrVBL4	.	.	.	.	.	.	.	.	.	.	(2)	(*N. greenwoodi*)	
MrVBL5	.	.	.	.	.	.	.	.	.	.	(2)	(*N. greenwoodi*)	
MrVBL6	.	.	.	.	.	.	.	.	.	.	(2)	(*N. greenwoodi*)	
MrVBL7	.	.	.	.	.	.	.	.	.	.	(2)	(*N. greenwoodi*)	
MrVBL8	.	.	.	.	.	.	.	.	.	.	(2)	(*N. greenwoodi*)	
MrVBL9	.	.	.	.	.	.	.	.	.	.	(2)	(*N. greenwoodi*)	
Mt1	.	.	.	.	.	.	.	.	.	.	(3)	(*N. greenwoodi*)	
Mt2	.	.	.	.	.	.	.	.	.	.	(3)	(*N. greenwoodi*)	
Mt3	.	.	.	.	.	.	.	.	.	.	(3)	(*N. greenwoodi*)	L=10
Mt4	.	.	.	.	.	.	.	.	.	.	(3)	(*N. greenwoodi*)	
Mt6	.	.	.	.	.	.	.	.	.	.	(3)	(*N. greenwoodi*)	
Py27	.	.	.	G	.	.	.	T	G	.	(5)	(*N. greenwoodi*)	
Py9	.	.	.	G	.	.	.	T	G	.	(5)	(*N. greenwoodi*)	
PyVbl1	.	K	.	.	.	.	.	.	.	.	(5)	(*N. greenwoodi*)	L=5
PyVBL2	.	.	.	.	.	.	.	.	.	.	(5)	(*N. greenwoodi*)	M2=6
PyVBL3	.	.	.	.	K	K	.	W	K	.	(5)	(*N. greenwoodi*)	
PyVBL5	.	.	.	S	K	K	.	T	G	.	(5)	(*N. greenwoodi*)	H=3
PyVBL6	.	.	.	S	K	K	.	T	G	.	(5)	(*N. greenwoodi*)	
Hic	G	.	S	.	K	G	.	T	G	.	(7)	(*N. greenwoodi*)	
Hid	.	.	.	.	G	G	.	T	G	.	(7)	(*N. greenwoodi*)	M2=5
Hie	.	.	.	.	K	G	.	T	G	.	(7)	(*N. greenwoodi*)	
HiVbl1	.	.	.	S	.	K	.	T	G	.	(7)	(*N. greenwoodi*)	H=5
HiVbl2	.	.	Y	.	K	G	.	T	G	.	(7)	(*N. greenwoodi*)	

Bw13	. . . S K K . T G .	(9)	(*N. greenwoodi*)	M1=1 M2=10 H=3
Bw16	. . . . G G . W K R	(9)	(*N. greenwoodi*)	
Bw2	. . . G . . . T G .	(9)	(*N. greenwoodi*)	
Bw32	. . . G . . . T G .	(9)	(*N. greenwoodi*)	
Bw33	. . . G . . . T G .	(9)	(*N. greenwoodi*)	
Bw39	. . . G . . . T G .	(9)	(*N. greenwoodi*)	
Bw4	. . . S K K . T G .	(9)	(*N. greenwoodi*)	
Ig	. . . S K K . T G .	(11)	(*N. greenwoodi*)	M2=5 H=17
Ig42	. . Y . G G . T G .	(11)	(*N. greenwoodi*)	
Ig43	. . . . G G . T G .	(11)	(*N. greenwoodi*)	
Ig44	. . . . G G . T G .	(11)	(*N. greenwoodi*)	
Ig45	. . . . G G . T G .	(11)	(*N. greenwoodi*)	
Ig46	. . C . G G . T G .	(11)	(*N. greenwoodi*)	
Ig47	. . . S K K . T G .	(11)	(*N. greenwoodi*)	
Ig48	. . . S K K . T G .	(11)	(*N. greenwoodi*)	
IgF	. . Y . G G . T G .	(11)	(*N. greenwoodi*)	
IgMB	. . . S K K . T G .	(11)	(*N. greenwoodi*)	
IgMC	. . . S K K . T G .	(11)	(*N. greenwoodi*)	
Se17	. . Y . G G . T G .	(13)	(*N. greenwoodi*)	M2=1 H=7
Se27	. . . . G G . T G .	(13)	(*N. greenwoodi*)	
Se33	. . . S K K . T G .	(13)	(*N. greenwoodi*)	
Se5	. . . . G G . T G .	(13)	(*N. greenwoodi*)	
So1	. . . . G G . T G .	(14)	(*N. greenwoodi*)	H=8
So15	. . . . G G . T G .	(14)	(*N. greenwoodi*)	
So3	. . . . G G . T G .	(14)	(*N. greenwoodi*)	
So69	. . . . G G . T G .	(14)	(*N. greenwoodi*)	
Nm1	. . C . G G . T G .	(15)	(*N. greenwoodi*)	H=50
Nm10	. . . . G G . T G .	(15)	(*N. greenwoodi*)	
Nm23	. . C . G G . T G .	(15)	(*N. greenwoodi*)	
Nm28	. . C . G G . T G .	(15)	(*N. greenwoodi*)	
Nm30	. . Y . G G . T G .	(15)	(*N. greenwoodi*)	
Nm31	. . C . G G . T G .	(15)	(*N. greenwoodi*)	
Nm5	. . . . G G . T G .	(15)	(*N. greenwoodi*)	
Nm7	. . C . G G . T G .	(15)	(*N. greenwoodi*)	
13846	. . C . G G . T G .	(15)	(*N. greenwoodi*)	
13851	. . C . G G . T G .	(15)	(*N. greenwoodi*)	
13852	. . C . G G . T G .	(15)	(*N. greenwoodi*)	
13853	. . Y . G G . T G .	(15)	(*N. greenwoodi*)	
13855	. . . . G G . T G .	(15)	(*N. greenwoodi*)	
13857	. . . . G G . T G .	(15)	(*N. greenwoodi*)	
13882	. . C . G G . T G .	(15)	(*N. greenwoodi*)	
13883	. . C . G G . T G .	(15)	(*N. greenwoodi*)	
13884	. . . . G G . T G .	(15)	(*N. greenwoodi*)	
13888	. . . . G G . T G .	(15)	(*N. greenwoodi*)	
13889	. . . . G G . T G .	(15)	(*N. greenwoodi*)	
13896	. . C . G G . T G .	(15)	(*N. greenwoodi*)	
13897	. . . . G G . T G .	(15)	(*N. greenwoodi*)	
13899	. . Y . G G . T G .	(15)	(*N. greenwoodi*)	
13900	. . Y . G G . T G .	(15)	(*N. greenwoodi*)	
13901	. . C . G G . T G .	(15)	(*N. greenwoodi*)	
13903	. . C . G G . T G .	(15)	(*N. greenwoodi*)	

Ma10	.	.	Y	.	G	G	.	T	G	.	(10)	(*N. omnicaeruleus*)
Ma11	.	.	.	.	G	G	.	T	G	.	(10)	(*N. omnicaeruleus*)
Ma12	.	.	C	.	G	G	.	T	G	.	(10)	(*N. omnicaeruleus*)
Ma13	.	.	Y	.	G	G	.	T	G	.	(10)	(*N. omnicaeruleus*)
Ma34	.	.	.	.	G	G	.	T	G	.	(10)	(*N. omnicaeruleus*)
MaA	.	.	C	.	G	G	.	T	G	.	(10)	(*N. omnicaeruleus*)
MaB	.	.	.	.	G	G	.	T	G	.	(10)	(*N. omnicaeruleus*)
MaC	.	.	.	.	G	G	.	T	G	.	(10)	(*N. omnicaeruleus*)
MaD	.	.	.	.	G	G	.	T	G	.	(10)	(*N. omnicaeruleus*)
MaE	.	.	.	.	G	G	.	T	G	.	(10)	(*N. omnicaeruleus*)
MaG	.	.	.	.	G	G	.	T	G	.	(10)	(*N. omnicaeruleus*)
MaH	.	.	.	.	G	G	.	T	G	.	(10)	(*N. omnicaeruleus*)
MaI	.	.	Y	.	G	G	.	T	G	.	(10)	(*N. omnicaeruleus*)
MaJ	.	.	Y	.	G	G	.	T	G	.	(10)	(*N. omnicaeruleus*)
MaK	.	.	.	.	G	G	.	T	G	.	(10)	(*N. omnicaeruleus*)
MaL	.	.	.	.	G	G	.	T	G	.	(10)	(*N. omnicaeruleus*)
MaM	.	.	.	.	G	G	.	T	G	.	(10)	(*N. omnicaeruleus*)
MaN	.	.	.	.	G	G	.	T	G	.	(10)	(*N. omnicaeruleus*)
MaO	.	.	Y	.	G	G	.	T	G	.	(10)	(*N. omnicaeruleus*)
MaP	.	.	.	.	G	G	.	T	G	.	(10)	(*N. omnicaeruleus*)
amino acid positions	44	150		168	177	179	230	275	275	282		
	I	I		A	S	F	T	C		T		
	M	A		G	A	V	A	I		A		

H=40
