# Supplementary material for: Divergent Selection on Opsins Drives Incipient Speciation in Lake Victoria Cichlids
Source: PLoS Biol. 2006 Dec 5;4(12):e433. doi: 10.1371/journal.pbio.0040433 (PMC1750929; doi:10.1371/journal.pbio.0040433)
Supplement: Figure S3 — The nucleotide sites are shown on top of the alignment. n and s indicate nonsynonymous and synonymous sites, respectively. Dots indicate where nucleotides are identical with those in the top line. The sequences of N. omnicaeruleus from Makobe (N. omnicaeruleus Ma) and N. greenwoodi from Marumbi (N. greenwoodi Mr) are aligned at the bottom. The sampling station numbers are described in Figure 1A. (52 KB PDF) [file pbio.0040433.sg003.pdf]

		nucleotide sites	139	370	465	529	535	664	679	688	823	824	826	891	Station	Number of sequences per allele group
		syn/non-syn	n	n	s	n	n	n	n	n	n	n	n	s		
M. mbipi	MaI		C	A	C	G	G	C	T	G	T	G	G	C	10	H=30
	MaII		.	.	.	.	.	.	.	.	.	.	.	.		
	MaIII		.	.	.	.	.	.	.	.	.	.	R	.		
	Ma15		.	.	.	.	.	.	.	.	.	.	R	.		
	Ma24		.	.	T	.	.	.	.	.	.	.	.	.		
	Ma8		.	.	T	.	.	.	.	.	.	.	.	.		
	Ma9		.	.	.	.	.	.	.	.	.	.	R	.		
	Ma2080		.	.	T	.	.	.	.	.	.	.	.	.		
	Ma2084		.	.	T	.	.	.	.	.	.	.	.	.		
	Ma2087		.	.	Y	.	.	.	.	.	.	.	R	.		
	Ma2088		.	.	T	.	.	.	.	.	.	.	.	.		
	Ma2089		.	.	T	.	.	.	.	.	.	.	.	.		
	Ma2090		.	.	T	.	.	.	.	.	.	.	.	.		
	Ma2093		.	.	T	.	.	.	.	.	.	.	.	.		
	Ma2110		.	.	T	.	.	.	.	.	.	.	.	.		
	Ig10		.	.	T	.	.	.	.	.	.	.	.	.	11	H=9, other=1
	Ig12		.	.	T	K	.	.	.	R	W	K	.	.		
	Ig52		G	.	T	.	.	.	.	.	.	.	.	.		
	Ig53		.	.	T	.	.	.	.	.	.	.	.	.		
	Ig54		.	.	T	.	.	.	.	.	.	.	.	.		
	Se39		.	.	T	.	.	.	.	.	.	.	.	.	13	H=2
	hi025		.	.	T	.	.	.	.	A	A	T	.	.	7	H=24, M3=8
	hi143		.	.	T	.	.	.	.	.	.	.	.	.		
	hi144		.	.	T	.	.	.	.	A	A	T	.	.		
	hi153		.	.	T	.	.	.	.	R	W	K	.	.		
	hi154		.	.	T	.	.	.	.	.	.	.	.	.		
	hi160		.	M	T	.	.	.	.	.	.	.	.	.		
	hi161		.	.	T	.	.	.	.	.	.	.	.	.		
	hi162		.	.	T	.	.	.	.	.	.	.	.	.		
	hi163		.	.	.	.	.	.	.	.	.	.	.	.		
	hi165		.	.	T	.	.	.	.	A	A	T	.	.		
	hi198		.	.	Y	.	.	.	.	.	.	.	.	.		
	hi200		.	.	.	.	.	.	.	.	.	.	.	.		
	HiI		.	.	T	.	.	.	.	.	.	.	.	.		
	HiIII		.	.	T	.	.	.	.	.	.	.	.	.		
	HiIIII		.	.	T	.	.	.	.	R	W	K	.	.		
	HiIIIII		.	.	T	.	.	.	.	.	.	.	.	.		
	Py10		.	.	.	.	.	.	.	.	.	.	.	.	5	H=9, M3=26, other=1
	Py100		.	.	T	.	.	.	.	A	A	T	.	.		
	Py118		.	.	T	.	.	.	.	A	A	T	.	.		
	Py139		.	.	T	.	.	.	.	A	A	T	.	.		
	Py151		.	.	T	.	.	M	W	R	W	K	.	Y		
	Py169		.	.	T	.	.	.	.	A	A	T	.	.		
	Py179		.	.	.	.	.	.	.	.	.	.	.	.		
	Py205		.	.	.	.	.	.	.	R	W	K	.	.		
	Py206		.	.	.	.	.	.	.	R	W	K	.	.		
	Py215		.	.	Y	.	.	.	.	R	W	K	.	.		
	Py216		.	.	T	.	.	.	.	A	A	T	.	.		
	Py231		.	.	Y	.	.	.	.	R	W	K	.	.		
	Py232		.	.	T	.	.	.	.	A	A	T	.	.		
	Py28		.	.	T	.	.	.	.	A	A	T	.	.		
	PyI		.	.	T	.	.	.	.	A	A	T	.	.		
	PyII		.	.	T	.	.	M	A	A	W	K	.	Y		
	PyIII		.	.	T	.	.	.	.	A	A	T	.	.		
	PyIIII		.	.	T	.	.	.	.	A	A	T	.	.		
N. omnicaeruleus	Ma		.	.	T	.	.	.	.	.	.	.	.	.		
N. greenwoodi	Mr		.	.	T	T	T	.	.	.	A	T	.	.		
