# Supplementary material for: Divergent Selection on Opsins Drives Incipient Speciation in Lake Victoria Cichlids
Source: PLoS Biol. 2006 Dec 5;4(12):e433. doi: 10.1371/journal.pbio.0040433 (PMC1750929; doi:10.1371/journal.pbio.0040433)
Supplement: Figure S4 — The nucleotide sites are shown on top of the alignment. n and s indicate nonsynonymous and synonymous sites, respectively. Dots indicate where nucleotides are identical with those in the top line. The sequences of N. omnicaeruleus from Makobe (N. omnicaeruleus Ma) and N. greenwoodi from Marumbi (N. greenwoodi Mr) are aligned at the bottom. The sampling station numbers are described in Figure 1A. (50 KB PDF) [file pbio.0040433.sg004.pdf]

								Station	Number of sequences per allele group
nucleotide sites		465	503	529	535	823	824		
syn/non-syn		s	n	n	n	n	n		
N. rufocaudalis	11720	T	C	G	G	T	G	10	H=10
	14574	.	.	.	.	.	.		
	12124	.	.	.	.	.	.		
	12125	.	.	.	.	.	.		
	14562	.	.	.	.	.	.		
	10982	.	.	.	.	.	.	17	M2=1<br>H=5
	10990	.	S	K	K	.	.		
	10988	.	.	.	.	.	.		
	10722	Y	.	.	.	.	.	6	H=16
	10726	.	.	.	.	.	.		
	10731	.	.	.	.	.	.		
	10733	.	.	.	.	.	.		
	10734	.	.	.	.	.	.		
	11296	.	.	.	.	.	.		
	11300	.	.	.	.	.	.		
	11329	.	.	.	.	.	.		
	11142	.	.	.	.	.	.	16	H=10
	11144	.	.	.	.	.	.		
	11158	.	.	.	.	.	.		
	11160	.	.	.	.	.	.		
	15184	.	.	.	.	.	.		
	14859	.	.	.	.	.	.	18	H=4
	15000	.	.	.	.	.	.		
	10085	.	.	.	.	.	.	5	L=2<br>H=40
	10747	.	.	.	.	.	.		
	10784	.	.	.	.	.	.		
	10795	.	.	.	.	.	.		
	10796	.	.	.	.	.	.		
	10798	.	.	.	.	.	.		
	11210	Y	.	.	.	.	.		
	11211	.	.	.	.	.	.		
	11223	.	.	.	.	.	.		
	11249	.	.	.	.	.	.		
	11251	.	.	.	.	.	.		
	11252	.	.	.	.	.	.		
	11269	.	.	K	K	W	K		
	11270	.	.	.	.	.	.		
	11271	.	.	.	.	.	.		
	11272	.	.	.	.	.	.		
	11273	.	.	.	.	.	.		
	11274	.	.	.	.	.	.		
	11275	.	.	K	K	W	K		
	11276	.	.	.	.	.	.		
	11278	.	.	.	.	.	.		
N. omnicaeruleus	Ma	.	.	.	.	.	.		
N. greenwoodi	Mr	.	.	T	T	A	T		
