# Supplementary material for: Divergent Selection on Opsins Drives Incipient Speciation in Lake Victoria Cichlids
Source: PLoS Biol. 2006 Dec 5;4(12):e433. doi: 10.1371/journal.pbio.0040433 (PMC1750929; doi:10.1371/journal.pbio.0040433)
Supplement: Figure S5 — The nucleotide sites are shown on top of the alignment. n and s indicate nonsynonymous and synonymous sites, respectively. Dots indicate where nucleotides are identical with those in the top line. The sequences of N. omnicaeruleus from Makobe (N. omnicaeruleus Ma) and N. greenwoodi from Marumbi (N. greenwoodi Mr) are aligned at the bottom. The sampling station numbers are described in Figure 1A. (64 KB PDF) [file pbio.0040433.sg005.pdf]

nucleotide sites		152	465	523	529	535	645	647	688	794	823	824	844	1022	Station	Number of sequences per allele group
syn/non-syn		n	s	n	n	n	s	n	n	n	n	n	n	n		
P. pundamilia	By17	A	T	G	G	G	G	T	A	G	A	T	A	G	1	P=2
	Mr10	.	.	.	.	.	.	.	.	.	.	.	.	.	2	P=10
	Mr12	.	.	.	.	.	.	.	.	.	.	.	.	.		
	Mr40	.	.	.	.	.	.	.	.	.	.	.	.	.		
	Mr41	.	.	.	.	.	.	.	.	.	.	.	.	.		
	Mr42	.	.	.	.	.	.	.	.	.	.	.	.	.		
	Lu21	.	.	.	.	.	.	.	.	.	.	.	.	.	4	P=8
	Lu30	.	.	.	.	.	.	.	.	.	.	.	.	.		
	Lu7	.	.	.	.	.	.	.	.	.	.	.	.	.		
	Lu9	.	.	.	.	.	.	.	.	.	.	.	.	.		
	Mt48	.	.	.	.	.	.	.	.	.	.	.	.	.	3	P=4
	Mt54	.	.	.	.	.	.	.	.	.	.	.	.	.		
	Py117	.	.	.	.	.	.	W	R	.	.	G	.	.	5	P=14 other=4
	Py122	.	.	.	.	.	.	.	.	.	.	.	.	.		
	Py123	.	.	.	K	K	.	W	R	.	.	.	.	.		
	Py20	.	.	.	.	.	.	.	.	.	.	.	.	.		
	Py58	.	.	.	.	.	.	.	.	.	.	.	.	.		
	Py59	.	.	.	.	.	.	.	.	.	.	.	.	.		
	Py63	.	.	.	.	.	.	.	.	.	.	.	.	.		
	Py64	.	.	.	.	.	.	.	.	.	.	.	.	.		
	Py65	.	.	.	.	.	.	.	.	.	.	.	.	.		
	Ks1	.	.	.	.	.	.	.	.	.	.	.	.	.	6	P=8
	Ks29	.	.	.	.	.	.	.	.	.	.	.	.	.		
	Ks2	.	.	.	.	.	.	.	.	.	.	.	.	.		
	Ks8	.	.	.	.	.	.	.	.	.	.	.	.	.		
	Bw6	.	.	R	.	.	S	W	.	.	.	.	R	.	9	P=1 other=5
	Bw7	.	.	.	.	.	C	A	R	R	W	K	R	.		
	Bw8	.	.	R	.	.	C	A	.	.	.	.	G	.		
	Ig13	.	.	.	.	.	.	.	.	.	.	.	.	R	11	P=2 other=4
	Ig39	.	Y	.	.	.	C	A	R	R	W	K	R	.		
	Ig40	.	.	.	.	.	C	A	.	.	.	.	G	.		
	Ju1	.	.	.	.	.	.	.	.	.	.	.	.	A	8	P=5 other=1
	Ju2	R	.	.	.	.	.	.	.	.	.	.	.	R		
	Ju30	.	.	.	.	.	S	W	.	R	.	.	R	R		
	Ma3411	.	.	.	.	.	.	.	.	.	.	.	.	.	10	P=10
	Ma341	.	.	.	.	.	.	.	.	.	.	.	.	.		
	Ma5893	.	.	.	.	.	.	.	.	.	.	.	.	.		
	Ma5	.	.	.	.	.	.	.	.	.	.	.	.	.		
	Ma92	.	.	.	.	.	.	.	.	.	.	.	.	.		
	Ru27	.	.	R	.	.	.	.	.	.	.	.	.	.	12	P=6 other=2
	Ru28	.	.	A	.	.	.	.	.	.	.	.	.	.		
	Ru29	.	.	A	.	.	.	.	.	.	.	.	.	.		
	Ru37	.	.	.	.	.	C	A	R	.	W	K	.	.		
N. omnicaeruleus	Ma	.	.	.	.	.	C	A	G	.	T	G	.	.		
N. greenwoodi	Mr	.	.	.	T	T	C	A	G	.	.	.	.	.		
