# Supplementary material for: Divergent Selection on Opsins Drives Incipient Speciation in Lake Victoria Cichlids
Source: PLoS Biol. 2006 Dec 5;4(12):e433. doi: 10.1371/journal.pbio.0040433 (PMC1750929; doi:10.1371/journal.pbio.0040433)
Supplement: Figure S6 — Maximum-likelihood analysis was performed with MOLPHY version 2.3 [44]. An NJ tree was used as the starting tree for a local rearrangement search for the ML tree with the HKY85 model [45]. The scale bar indicates the number of substitutions per site. Bootstrap values are shown at the branches. An alignment of all informative sites of alleles and the frequencies (%) of each allele are shown in right panel. Dots indicate where nucleotides are identical with those in the top line. The frequency of “other” alleles is not shown. (41 KB PDF) [file pbio.0040433.sg006.pdf]

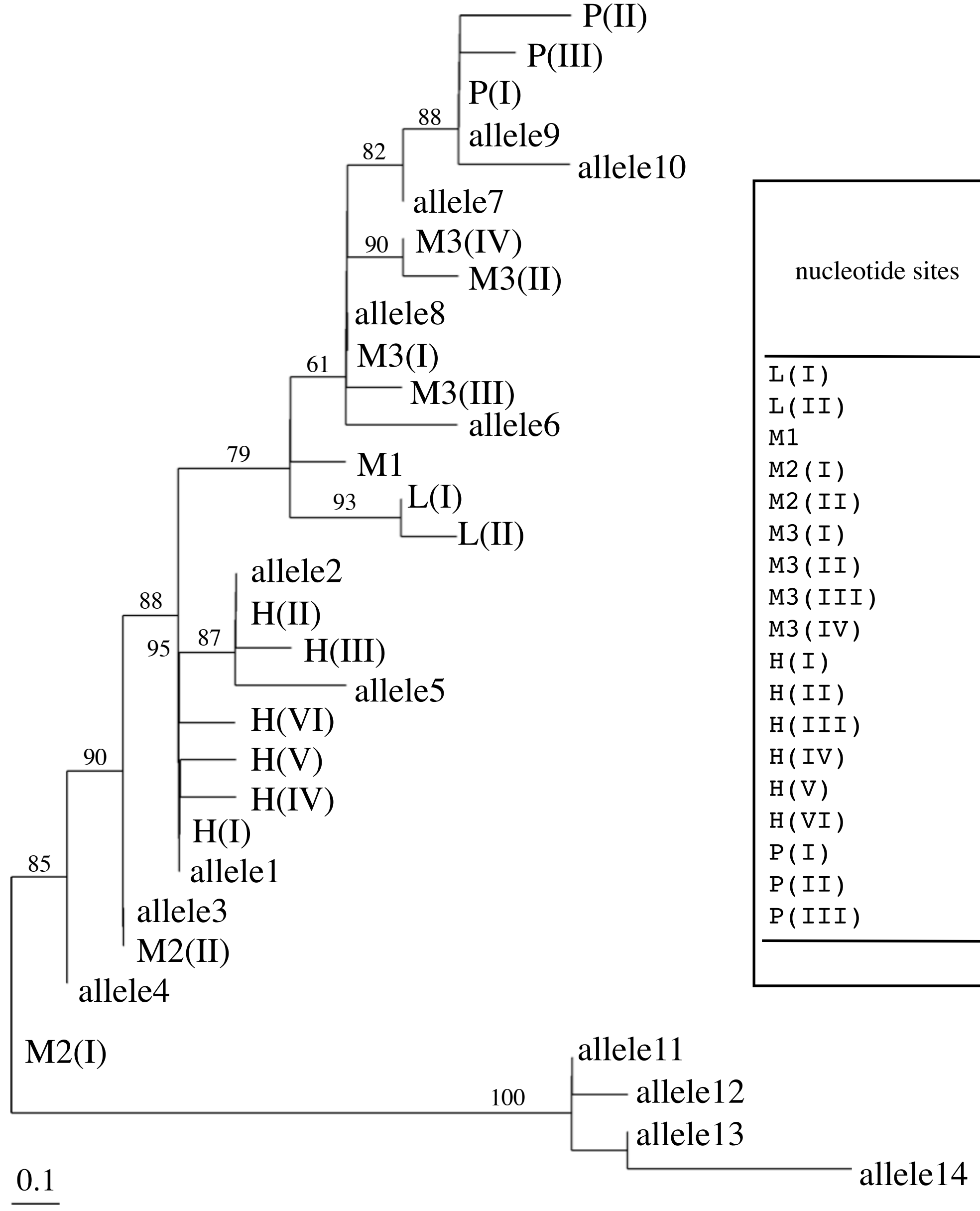

Haplotype	Nucleotide sites<br>1<br>1344555566666888880<br>3746022344678222492<br>9095339557498346412	*N. greenwoodi*	*N. rufocaudalis*	*M. mbipi*	*P. pundamilia*
L(I)	CATTCGTTCACTGATGACG	30	2		
L(II)	..G................	0.4			
M1	......GG........G..	0.4			
M2(I)	....G........TG....	10			
M2(II)	.......G.....TG....	1	1		
M3(I)	......GG....A......			22	
M3(II)	......GG..AAA......			1	
M3(III)	...C..GG....A......			2	
M3(IV)	......GG...AA......			1	
H(I)	......GG.....TG....	38	94	49	
H(II)	...C..GG.....TG....	19	3	18	
H(III)	...C..GG.....TGA...			4	
H(IV)	G.....GG.....TG....			2	
H(V)	.C....GG.....TG....			1	
H(VI)	......GG.....TG..T.			1	
P(I)	......GGGT..A......				67
P(II)	......GGGT..A.....A				5
P(III)	.....AGGGT..A......				6
