# Supplementary material for: Divergent Selection on Opsins Drives Incipient Speciation in Lake Victoria Cichlids
Source: PLoS Biol. 2006 Dec 5;4(12):e433. doi: 10.1371/journal.pbio.0040433 (PMC1750929; doi:10.1371/journal.pbio.0040433)
Supplement: Figure S8 — (15 KB PDF) [file pbio.0040433.sg008.pdf]

LWSB_LF: 5’-CCTCACTCTTCTGATTTATTCACAGCTCAT-3’
LWSB_F1: 5’-AACTCTGTTAACCCTTTGCAA-3’
LWSB_F2: 5’-TGTGGAGGAACCTGAGTTGT-3’
LWSB_F3: 5’-AACATTGTTGCTGCCACTGA-3’
LWSB_F4: 5’-TGTTAACGTGTGAAGTTGGATTTC-3’
LWSB_F5: 5’-CAGTTCAGGGTTATTGCTTTGC-3’
LWS_F1: 5’-GCGGTATCATGAAGATTCAACCA-3’
LWS_F4: 5’-TTGCTGCAAGGCGGTATCAT-3’
LWS_F7: 5’-CTGTGGTGCTGTTAGTGATTCTG-3’
LWS_F8: 5’-AAGTCTCCAGGATGGTCGTTGT-3’
LWSB_F8: 5’-AATGAAACAGCAGCAAGGCTA-3’
LWSB_F9: 5’-ATGACACAGCATGGATGACAG-3’
LWSB_F10: 5’-AGCTTCAATAGTCGTCCTCTTTG-3’
LWSB_F11: 5’-GGATGAGACACAAAAGATCAAC-3’
LWSB_F12: 5’-GTCAACAACTGAAACCTGCACT-3’
LWSB_F15: 5’-CAATGTCTGACTGTGATTAGTTCA-3’
LWSB_R1: 5’-CTTCTCTCCTGCTGTACAGATGT-3’
LWSB_R2: 5’-CCGTGAATGTCTAACCTATTTGAC-3’
LWSB_R3: 5’-CACTGTGTGTTACTAGCAAAGCA-3’
LWSB_R4: 5’-CTGAACGCAATCTGTCTAAAGTC-3’
LWSB_R5: 5’-GCTTGGTGCTACAGATCTGACT-3’
LWS_R1: 5’-GGAGACTTCGGAGCCATCATC-3’
LWS_R4: 5’-CAGTCCATGAGGCCAGTACCTA-3’
LWSB_R8: 5’-GAAATCTGACACTAGCTCACTGCT-3’
LWSB_R9: 5’-CAATACTGTCCTATGTTGGCAA-3’
LWSB_R10: 5’-GAACAGCAAACACTACCAGAATG-3’
LWSB_R12: 5’-CATCCTCACAAATCAGATTAGCA-3’
LWSB_R13: 5’-TGTGGATCTTTCACTGGCTACA-3’
LWSB_R14: 5’-CGATAGTCAAATGTCTGAGTCAGT-3’
LWSB_LR: 5’-TCATTGTGGCATCACTGGTCTAGTCA-3’
Eco6LWS: 5’-CACAGAATTCGCCACCATGGCAGAAGAGTGGGGAAAACAAAGTT-3’
LWS_Flag_stop_NotI: 5’-CACAGCGGCCGCTTACTTGTCATCGTCGTCCTTGTAGTCTGCGGGAGCCACAGAGGAGACCT-3’
