# Supplementary material for: Divergent Selection on Opsins Drives Incipient Speciation in Lake Victoria Cichlids
Source: PLoS Biol. 2006 Dec 5;4(12):e433. doi: 10.1371/journal.pbio.0040433 (PMC1750929; doi:10.1371/journal.pbio.0040433)
Supplement: Table S1 — (81 KB PDF) [file pbio.0040433.st001.pdf]

Table S1. F_{ST} values for polymorphic neutral markers

populations**	polymorphic loci*			
	1801	1918	1807	average (S. E.)
Marumbi-Namatembi	0.0420	0.0400	0.0460	0.043±0.0024
Marumbi-Makobe	0.0006	0.0920	0.0037	0.032±0.042
Namatembi-Makobe	0.0340	0.0120	0.0036	0.017±0.013

*SINE insertion polymorphic loci [1]

**The number of individuals; *N. greenwoodi*: n=29 at Marumbi, n=25 at Namatembi; *N. omnicaeruleus*, n=20 at Makobe

1.Terai Y, Takezaki N, Mayer WE, Tichy H, Takahata N, et al. (2004) Phylogenetic relationships among East African haplochromine fishes as revealed by short interspersed elements (SINEs). J Mol Evol 581: 64-78.
