# Supplementary material for: Divergent Selection on Opsins Drives Incipient Speciation in Lake Victoria Cichlids
Source: PLoS Biol. 2006 Dec 5;4(12):e433. doi: 10.1371/journal.pbio.0040433 (PMC1750929; doi:10.1371/journal.pbio.0040433)
Supplement: Table S2 — (34 KB PDF) [file pbio.0040433.st002.pdf]

Table S2. HKA test for the statistical significance (p) of heterogeneity between regions

population	outgroup	regions		
		upstream-LWS gene	downstream-LWS gene	upstream-downstream
Marumbi	Makobe	0.0379*	0.1099	0.6817
Marumbi	Namatembi	0.0303*	0.0561	0.8342
Makobe	Marumbi	0.0043**	0.0010**	0.8342
Makobe	Namatembi	0.1996	0.1806	0.9029
Namatembi	Marumbi	0.0024**	0.0004***	0.5592
Namatembi	Makobe	0.6369	0.5577	0.3688

*0.01<p<0.05, **0.001<p<0.01, ***p<0001

The up and downstream regions, described in Figure 2, are used as neutral regions. The p values indicate the statistical significance for the comparison of the ratio of polymorphism within a population to divergence from outgroup (single sequence from outgroup population) between the *LWS* gene region (test region) and up and downstream regions (neutral regions).
